# Peste des Petits Ruminants Virus Nucleocapsid Protein Inhibits Beta Interferon Production by Interacting with IRF3 To Block Its Activation

**DOI:** 10.1128/JVI.00362-19

**Published:** 2019-07-30

**Authors:** Zixiang Zhu, Pengfei Li, Fan Yang, Weijun Cao, Xiangle Zhang, Wen Dang, Xusheng Ma, Hong Tian, Keshan Zhang, Miaotao Zhang, Qinghong Xue, Xiangtao Liu, Haixue Zheng

**Affiliations:** a State Key Laboratory of Veterinary Etiological Biology, National Foot and Mouth Diseases Reference Laboratory, Key Laboratory of Animal Virology of Ministry of Agriculture, Lanzhou Veterinary Research Institute, Chinese Academy of Agricultural Sciences, Lanzhou, China; b College of Veterinary Medicine, Northwest A&F University, Yangling, Shaanxi, China; c China Institute of Veterinary Drug Control, Beijing, China; Hudson Institute of Medical Research

**Keywords:** IRF3, PPRV, immune suppression, nuclear translocation, nucleocapsid

## Abstract

Peste des petits ruminants virus (PPRV) is the etiological agent of peste des petits ruminants, causing acute immunosuppression in its natural hosts. However, the molecular mechanisms by which PPRV antagonizes the host immune responses have not been fully characterized. In particular, how PPRV suppresses the activation of the host RIG-I-like receptor (RLR) pathway has yet to be clarified. In this study, we demonstrated that PPRV infection significantly suppresses RLR pathway activation and type I interferon (IFN) production and identified PPRV N protein as an extremely important antagonistic viral factor that suppresses beta interferon (IFN-β) and IFN-stimulated gene (ISG) expression. A detailed analysis showed that PPRV N protein inhibited type I IFN production by targeting interferon regulatory factor 3 (IRF3), a key molecule in the RLR pathway required for type I IFN induction. PPRV N protein interacted with IRF3 (but not with other components of the RLR pathway, including MDA5, RIG-I, VISA, TBK1, and MITA) and abrogated the phosphorylation of IRF3. As expected, PPRV N protein also considerably impaired the nuclear translocation of IRF3. The TBK1-IRF3 interaction was involved significantly in IRF3 phosphorylation, and we showed that PPRV N protein inhibits the association between TBK1 and IRF3, which in turn inhibits IRF3 phosphorylation. The amino acid region 106 to 210 of PPRV N protein was determined to be essential for suppressing the nuclear translocation of IRF3 and IFN-β production, and the 140 to 400 region of IRF3 was identified as the crucial region for the N-IRF3 interaction. Together, our findings demonstrate a new mechanism evolved by PPRV to inhibit type I IFN production and provide structural insights into the immunosuppression caused by PPRV.

**IMPORTANCE** Peste des petits ruminants is a highly contagious animal disease affecting small ruminants, which threatens both small livestock and endangered susceptible wildlife populations in many countries. The causative agent, peste des petits ruminants virus (PPRV), often causes acute immunosuppression in its natural hosts during infection. Here, for the first time, we demonstrate that N protein, the most abundant protein of PPRV, plays an extremely important role in suppression of interferon regulatory factor 3 (IRF3) function and type I interferon (IFN) production by interfering with the formation of the TBK1-IRF3 complex. This study explored a novel antagonistic mechanism of PPRV.

## Abstract (precis)

Peste des petits ruminants is a highly contagious animal disease affecting small ruminants, which threatens both small livestock and endangered susceptible wildlife populations in many countries. The causative agent, peste des petits ruminants virus (PPRV), often causes acute immunosuppression in its natural hosts during infection. Here, for the first time, we demonstrate that N protein, the most abundant protein of PPRV, plays an extremely important role in suppression of interferon regulatory factor 3 (IRF3) function and type I interferon (IFN) production by interfering with the formation of the TBK1-IRF3 complex. This study explored a novel antagonistic mechanism of PPRV.

## INTRODUCTION

Peste des petits ruminants (PPR) is an acute contagious disease that mainly affects small domestic and wild ruminants, thereby threatening food security and the sustainable livelihoods of farmers across Africa, the Middle East, and Asia (1). The range of the disease has expanded in recent years, and outbreaks of PPR are causing great economic losses (2, 3). The causative agent, peste des petits ruminants virus (PPRV), belongs to the genus *Morbillivirus* in the family *Paramyxoviridae*. The viral genome is a negative single-stranded RNA, which encodes six structural proteins, the nucleocapsid (N), phosphoprotein (P), matrix (M), fusion (F), hemagglutinin (H), and polymerase (L) proteins, and three nonstructural proteins, C, W, and V (4). These viral proteins perform multiple roles in the pathogenicity of PPRV and can block the functions of various host proteins to counteract host antiviral responses.

A strong immune response and a large amount of neutralizing antibodies can suppress PPRV replication and quickly clear a PPRV infection (5, 6). However, PPRV infections have been known to be associated with acute immunosuppression in its natural hosts, which ensures successful infection and viral replication. Viral proteins are responsible for the suppression of host immune responses and contribute to viral pathogenesis via various strategies (4, 7), such as the induction of apoptosis (8) and suppression of antibody responses (9). The disruption of innate immune responses by PPRV has not been fully investigated, and only a limited number of reports have explored the mechanisms underlying the PPRV-induced suppression of the host innate immune system.

Our previous study suggested that PPRV infection suppresses host innate immune responses. The V protein of PPRV was identified as an important factor, blocking the host innate immune response by abrogating the nucleus translocation of STAT1/2 proteins (10). Our data indicated that the N and P proteins also suppress host immune response by blocking RIG-I-like receptor (RLR) pathway activation. However, the detailed mechanisms are unknown. N protein is the nucleocapsid protein of PPRV, with a molecular weight of 58 kDa. As the most abundant viral protein in PPRV-infected cells, N protein has been investigated in detail and used extensively in the development of diagnostic products (4). Measles virus (MV) also belongs to the genus *Morbillivirus*, and the N protein of MV plays a significant role in the induction of immunosuppression by arresting cell proliferation (11, 12). The PPRV N protein binds to murine CD32 and inhibits inflammatory immune responses in mice (7). These reports suggest that N protein plays important roles in *Morbillivirus*-induced immunosuppression.

Understanding the factors that influence viral replication can provide a basis for devising control strategies for PPR. To better understand the role of PPRV N protein in the suppression of the RLR pathway-mediated antiviral response, we investigated the effect of N protein on type I IFN production. We found that PPRV N protein significantly inhibited beta interferon (IFN-β) and interferon-stimulated gene (ISG) expression. We identified the transcription factor IFN regulatory factor 3 (IRF3) as a target protein of N protein. N protein interacted with IRF3 and blocked the interaction between TBK1 and IRF3, which was crucial for the activation of IRF3 and the production of type I IFN. In this study, we identified the inhibitory activity of PPRV N protein on type I IFN production and thus demonstrated the suppressive role of PPRV N protein on the host innate immune system. We explored a novel antagonistic mechanism of PPRV.

## RESULTS

### PPRV N protein blocks type I IFN production and ISG expression.

**FIG 1:**
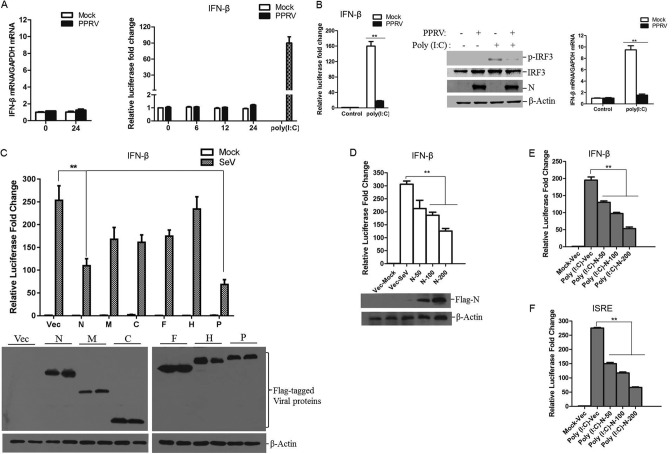

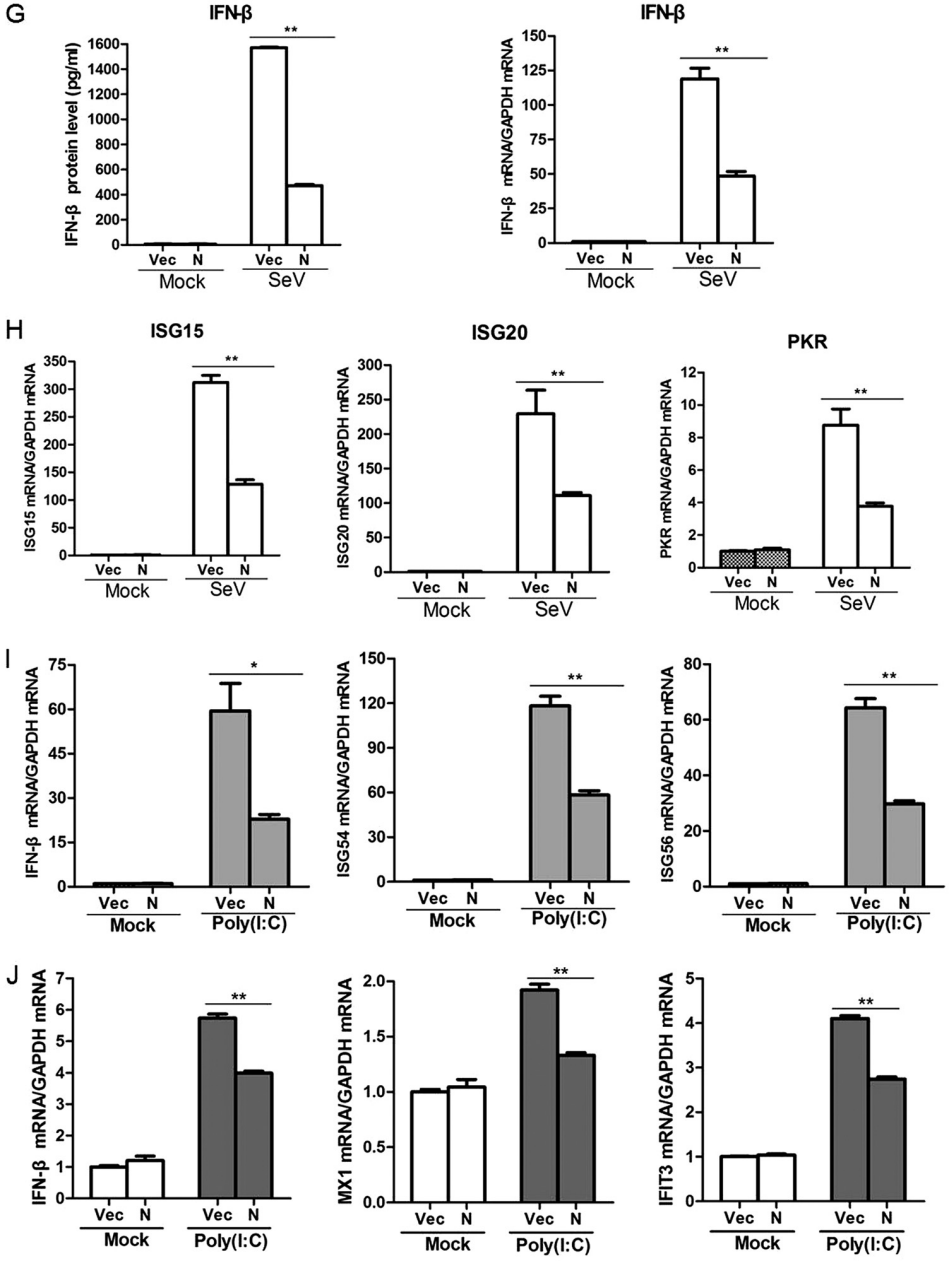
PPRV N protein suppresses IFN-β promoter activation and IFN-β production. (A) Goat fibroblasts were mock infected or infected with PPRV for 24 h, and then the mRNA expression of IFN-β was measured by qPCR (left). HEK-293T cells were cotransfected with an IFN-β promoter-driven reporter plasmid and pRL-TK plasmid for 24 h and then mock infected or infected with PPRV for 0, 6, 12, or 24 h. As a positive control, 24 h later, the cells were transfected with poly(I·C) for 24 h. The luciferase activity was then measured with a dual-luciferase assay (right). (B) HEK-293T cells were cotransfected with an IFN-β promoter-driven reporter plasmid and pRL-TK plasmid and mock infected or infected with PPRV before poly(I·C) transfection. After infection with PPRV for 24 h, the cells were transfected with poly(I·C) for another 24 h, and the luciferase activity was then measured (left). Goat fibroblasts were infected with PPRV for 24 h and then transfected with or without poly(I·C) for another 24 h; the expression of IRF3, phosphorylated IRF3 (p-IRF3), PPRV N protein, and β-actin was detected by Western blotting (middle). The mRNA expression of IFN-β was measured by qPCR (right). (C) HEK-293T cells were cotransfected with the indicated plasmids expressing various PPRV proteins and IFN-β promoter-driven reporter plasmids for 24 h and then infected with SeV for another 16 h. The luciferase activity was measured with a dual-luciferase assay. Expression of the viral proteins was evaluated by Western blotting. (D) HEK-293T cells were cotransfected with empty vector or increasing amounts of Flag-N-expressing plasmids (50, 100, or 200 ng) and IFN-β promoter-driven reporter plasmids for 24 h and then infected with SeV for another 16 h. The luciferase activity was then measured. Expression of N protein was detected by Western blotting. (E) HEK-293T cells were cotransfected with empty vector or increasing amounts of Flag-N-expressing plasmids (50, 100, or 200 ng) and IFN-β promoter-driven reporter plasmids for 24 h and then transfected with poly(I·C) for another 24 h. The luciferase activity was then measured. (F) HEK-293T cells were cotransfected with empty vector or increasing amounts of Flag-N-expressing plasmids (50, 100 or 200 ng) and ISRE reporter plasmids for 24 h, and the cells were then transfected with poly(I·C) for another 24 h; the luciferase activity was then measured. HEK-293T cells were transfected with empty vector- or Flag-N-expressing plasmids for 24 h and then mock infected or infected with SeV for 16 h. (G) IFN-β in the harvested supernatants was detect with an ELISA. (G and H) mRNA expression levels of IFN-β and ISGs (ISG15, ISG20, and PKR) in the collected cells were detected by qPCR. (I) HEK-293T cells were transfected with empty vector- or Flag-N-expressing plasmids for 24 h and then mock transfected or transfected with poly(I·C) for another 24 h. The mRNA expression levels of IFN-β and ISGs (ISG54 and ISG56) were determined by qPCR. (J) Goat fibroblasts were transfected with empty vector- or Flag-N-expressing plasmids for 24 h and then mock transfected or transfected with poly(I·C) for another 24 h. The mRNA expression levels of IFN-β and ISGs (MX1 and IFIT3) were determined by qPCR.

Type I IFN production is significantly involved in host antiviral processes. To investigate the interaction between PPRV and host type I IFN production, goat fibroblasts were mock infected or infected with PPRV, and the expression of IFN-β was measured. No significant change in IFN-β mRNA expression level was observed after PPRV infection (Fig. 1A). A luciferase reporter assay was also performed to evaluate the IFN-β production in PPRV-infected cells. HEK-293T cells were cotransfected with an IFN-β promoter-driven luciferase reporter plasmid and the internal control pRL-TK reporter plasmid, and the transfected cells were followed by mock infection or infection with PPRV. Polyriboinosinic polyribocytidylic acid [poly(I·C)], a synthetic analogue of double-stranded RNA, was also used to activate the IFN-β promoter-driven luciferase reporter system and referred as a control. Poly(I·C) strongly activated the IFN-β promoter. In contrast, IFN-β promoter-driven luciferase activity was scarcely detectable in the cells infected by PPRV (Fig. 1A). This suggested that PPRV infection does not induce IFN-β production and/or it considerably inhibits IFN-β production.

To investigate whether PPRV infection inhibited type I IFN production, HEK-293T cells were cotransfected with an IFN-β promoter-driven luciferase reporter plasmid and the pRL-TK reporter plasmid. The transfected cells were mock infected or infected with PPRV for 24 h and subsequently treated with or without poly(I·C). Transfection with poly(I·C) significantly induced the promoter activation of IFN-β in the mock-infected cells. However, this activity was remarkably decreased in the PPRV-infected cells (Fig. 1B), suggesting a suppressive effect of PPRV infection on type I IFN production. We also evaluated this suppressive effect of PPRV in goat fibroblasts. Goat fibroblasts were infected with PPRV for 24 h and then transfected with or without poly(I·C). Transfection with poly(I·C) significantly induced the phosphorylation of IRF3 and initiated the mRNA expression of IFN-β in the mock-infected cells. However, in the PPRV-infected cells, the poly(I·C)-induced phosphorylation of IRF3 and IFN-β production were significantly blocked (Fig. 1B). These results suggested that PPRV infection inhibits the activation of the RLR pathway and IFN-β production.

To identify the viral proteins that are involved in the inhibition of type I IFN production, HEK-293T cells were transfected with plasmids expressing various Flag-tagged viral proteins, the IFN-β promoter luciferase reporter plasmid, and the pRL-TK reporter plasmid and then infected with Sendai virus (SeV) (a strong and widely used activator of the type I IFN pathway). As shown in Fig. 1C, PPRV N and P proteins significantly inhibited SeV-induced IFN-β promoter activity. Because N protein is the most abundant viral protein in PPRV-infected cells, further experiments were performed to investigate the antagonistic role of N protein. A dose dependence assay was performed to confirm the antagonistic activity of N protein, which indicated that N protein blocked the SeV-induced IFN-β promoter activity in a dose-dependent manner (Fig. 1D). N protein similarly suppressed the poly(I·C)-induced IFN-β promoter activity in a dose-dependent manner (Fig. 1E). The suppressive effect of N protein on the poly(I·C)-induced activity of IFN-stimulated response element (ISRE) was evaluated and consistent with our earlier finding, suggesting that N protein blocks the activation of type I IFN production (Fig. 1F).

It is well known that infection by various RNA viruses activates the RLR pathway and initiates the expression of IFN-β and a set of ISGs (13). To confirm that PPRV N protein suppresses type I IFN production, the effect of PPRV N on IFN-β production and ISG expression was investigated further. HEK-293T cells were transfected with Flag-N-encoding plasmids and then infected with SeV. The cell supernatants and cells were collected separately for the enzyme-linked immunosorbent assay (ELISA) and quantitative PCR (qPCR) detection of IFN-β expression. The overexpression of N protein significantly suppressed SeV-induced mRNA expression and secretion of IFN-β, indicating that PPRV N protein blocked IFN-β production (Fig. 1G). The expression levels of some ISGs, including ISG15, ISG20 and PKR, were also measured with qPCR. The results showed that N protein significantly reduced the SeV-induced expression of ISG15, ISG20, and PKR mRNAs (Fig. 1H). The suppressive effect of N protein on poly(I·C)-induced IFN-β and ISG (ISG54 and ISG56) expression was also evaluated in HEK-293T cells and suggested that N protein inhibits poly(I·C)-induced IFN-β and ISG expression (Fig. 1I). We performed the same experiment in primary goat fibroblasts, which showed that poly(I·C)-induced expression of IFN-β and ISGs (MX1 and IFIT3) in the goat fibroblasts was also impaired by overexpression of PPRV N protein (Fig. 1J). These results confirmed that PPRV N protein inhibited IFN-β production and ISG expression.

### IRF3 might be the potential target of PPRV N protein.

**FIG 2:**
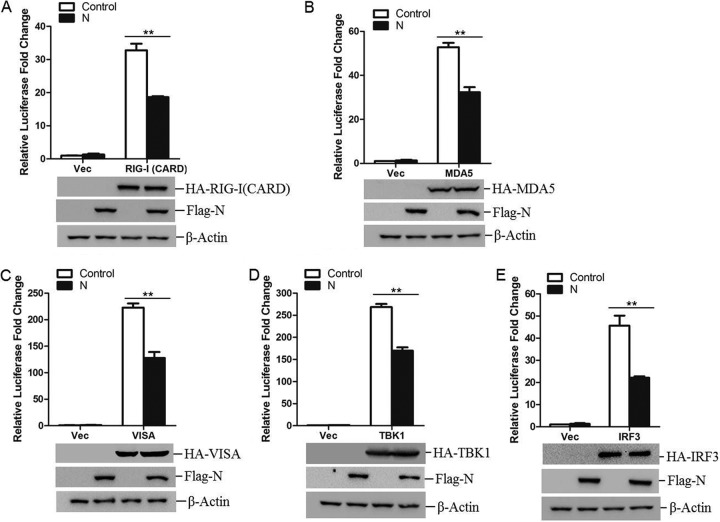
Effects of PPRV N protein on IFN-β promoter activation induced by various adaptor molecules. HEK-293T cells were cotransfected with empty vector- or Flag-N-expressing plasmids and the indicated plasmids expressing RIG-I(CARD) (A), MDA5 (B), VISA (C), TBK1 (D), or IRF3 (E), together with IFN-β promoter-driven reporter plasmids. The luciferase activity was measured at 24 hpt. Expression of various adaptor molecules and PPRV N protein was evaluated by Western blotting.

Induction of type I IFN is mainly mediated by RIG-I-like receptors (RLRs) during infection by RNA virus (14). The observed blockade of type I IFN production by PPRV N protein raises the possibility that the N protein targets one or several adaptors of the RLR signaling pathway. To investigate this possibility, HEK-293T cells were cotransfected with Flag-N-encoding plasmid and the plasmids expressing a set of adaptor molecules, including RIG-I(CARD), MDA5, VISA, TBK1, and IRF3, together with the IFN-β promoter-driven luciferase reporter plasmid and the internal control pRL-TK reporter plasmid. Luciferase activity was detected and analyzed at 26 h posttransfection (hpt). The overexpression of any of these adaptor molecules significantly activated IFN-β promoter activity. Interestingly, the expression of N protein suppressed activation of the IFN-β promoter induced by all of these adaptor molecules (Fig. 2A to E). Therefore, we speculated that the PPRV N protein targeted IRF3 to block type I IFN production.

### PPRV N protein interacts with IRF3.

**FIG 3:**
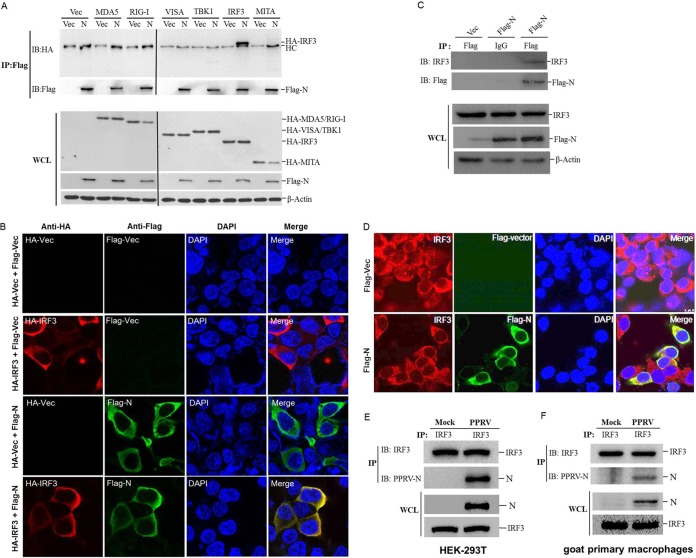
PPRV N protein interacts with both exogenous and endogenous IRF3. (A) HEK-293T cells were cotransfected with empty vector- or Flag-N-expressing plasmids and the indicated plasmids expressing different HA-tagged adaptor molecules for 48 h. The cells were then lysed and immunoprecipitated with an anti-Flag antibody. The whole-cell lysates (WCL) and immunoprecipitation (IP) complexes were analyzed by Western blotting using an anti-HA, anti-Flag, or anti-β-actin antibody. HC, heavy chain. (B) HEK-293T cells were cotransfected with HA empty vector plasmids or an HA-IRF3-expressing plasmid and Flag empty vector- or Flag-N-expressing plasmids for 36 h. The subcellular localization of HA-tagged IRF3 and Flag-tagged N protein was analyzed by IFA. (C) HEK-293T cells were transfected with empty vector- or Flag-N-expressing plasmids for 48 h. The cells were lysed and immunoprecipitated with an anti-Flag antibody or mouse normal IgG antibody. The WCL and IP complexes were analyzed by Western blotting using anti-IRF3, anti-Flag, or anti-β-actin antibodies. (D) HEK-293T cells were transfected with empty vector- or Flag-N-expressing plasmids for 36 h. The colocalization of endogenous IRF3 and Flag-tagged N protein was analyzed by IFA. (E and F) HEK-293T or goat primary macrophages cells were mock infected or infected with PPRV for 48 h. The cells were lysed and immunoprecipitated with an anti-IRF3 antibody. The WCL and IP complexes were analyzed by Western blotting using anti-IRF3 and anti-N antibodies.

To explore a possible interaction between N protein and the adaptor molecules of the RLR signaling pathway, HEK-293T cells were cotransfected with Flag-vector- or Flag-N-expressing plasmids and hemagglutinin (HA)-tagged MDA5-, RIG-I-, VISA-, TBK1-, IRF3-, or MITA-expressing plasmids. The co-immunoprecipitation (Co-IP) assay was performed with an anti-Flag antibody. As shown in Fig. 3A, IRF3 coprecipitated with N protein, whereas the other components of the RLR signaling pathway did not coprecipitate with N protein, suggesting that there was a direct interaction between PPRV N protein and IRF3. Because IRF3 plays an extremely important role in RLR pathway activation and the induction of IFN in response to infection by RNA viruses (15, 16), and PPRV N protein showed a significant suppressive effect on IRF3 and the molecules upstream from it (Fig. 2), we concentrated our subsequent investigations on IRF3.

To test whether PPRV N protein and IRF3 share similar subcellular locations, we conducted an indirect immunofluorescence microscopy assay (IFA). HEK-293T cells were cotransfected with Flag-vector- or Flag-N-expressing plasmids and HA-IRF3- or HA-vector-expressing plasmids, and the subcellular localization of N and IRF3 was investigated. As shown in Fig. 3B, both Flag-tagged N protein and HA-tagged IRF3 showed a cytoplasmic distribution, and the two proteins colocalized significantly in the cytoplasm. To confirm the interaction between N protein and endogenous IRF3, Co-IP and IFA were performed by transfecting HEK-293T cells with Flag-vector- or Flag-N-expressing plasmids, and the anti-IRF3 antibody was used to detect and visualize IRF3 expression. As shown in Fig. 3C and D, N protein immunoprecipitated endogenous IRF3, and N protein and endogenous IRF3 also colocalized in the cytoplasm. The interaction between PPRV N protein and cellular IRF3 in the context of viral infection was investigated by performing an immunoprecipitation experiment, which showed conclusively that IRF3 immunoprecipitated N protein in PPRV-infected HEK-293T cells and in goat primary macrophages (Fig. 3E and F). Similarly, the interaction of endogenous IRF3 and PPRV N protein in the goat fibroblasts was also confirmed (data not shown). These results firmly confirmed the interaction between PPRV N protein and the host IRF3 protein.

### PPRV N protein suppresses IRF3 phosphorylation.

**FIG 4:**
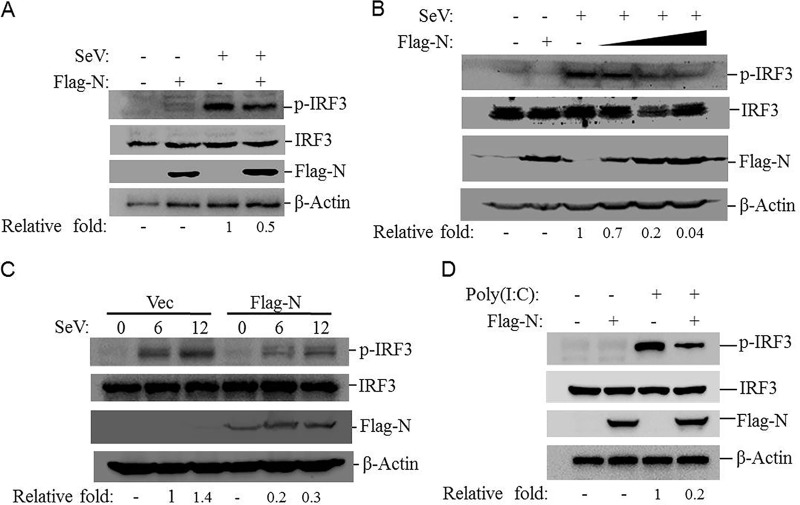
PPRV N protein inhibits SeV- and poly(I·C)-induced IRF3 phosphorylation. (A) HEK-293T cells were transfected with empty vector- or Flag-N-expressing plasmids for 24 h and then infected with SeV for 16 h. Expression of IRF3, phosphorylated IRF3 (p-IRF3), and N protein was detected by Western blotting. (B) HEK-293T cells were transfected with empty vector- or Flag-N-expressing plasmids (2 μg) for 24 h and then mock infected for 16 h (first and second lanes). HEK-293T cells were transfected with empty vector or increasing amounts of Flag-N-expressing plasmids (0.5, 1, or 2 μg) for 24 h (the empty vector was used in the whole transfection process to ensure that the cells received the same amounts of total plasmid). The cells were infected with SeV for 16 h (third through sixth lanes). All the cells were collected and subjected to Western blotting. Expression of IRF3, p-IRF3, and N protein was detected with the appropriate antibodies. (C) HEK-293T cells were transfected with equal amounts of empty vector or Flag-N-expressing plasmids for 24 h and then infected with SeV for 0, 6, or 12 h. Expression of IRF3, p-IRF3, and N protein was detected by Western blotting. The abundance of p-IRF3 was determined by densitometric analysis and normalized to β-actin. The relative fold change of the p-IRF3 expression level was determined. (D) HEK-293T cells were transfected with empty vector- or Flag-N-expressing plasmids for 24 h and then transfected with poly(I·C) for 24 h. Expression of IRF3, p-IRF3, and N protein was detected by Western blotting.

The phosphorylation of IRF3 is required for the activation of the RLR pathway and the induction of IFNs (17). PPRV N protein blocks type I IFN production by targeting IRF3. To investigate whether N protein affected the phosphorylation of IRF3, the levels of SeV-induced IRF3 phosphorylation in the absence or presence of N protein were investigated. HEK-293T cells were transfected with Flag-N- or empty vector-expressing plasmids and then infected with SeV. SeV infection induced significant phosphorylation of IRF3 in both Flag-N- and vector plasmid-transfected cells. However, the amount of phosphorylated IRF3 was markedly lower in the Flag-N-transfected cells than in the vector-transfected cells (Fig. 4A). A dose-dependent assay was then performed, which suggested that N protein inhibited SeV-induced phosphorylation of IRF3 in a dose-dependent manner (Fig. 4B). A time course assay was also performed. HEK-293T cells were transfected with Flag-vector- or Flag-N-expressing plasmids for 24 h, and the transfected cells were then infected with SeV. The cells were collected at different time points, and the expression level of phosphorylated IRF3 was detected by Western blotting. The results showed that overexpression of N protein suppressed SeV-induced phosphorylation of IRF3 (Fig. 4C). The effect of N protein on poly(I·C)-induced phosphorylation of IRF3 was also investigated, which showed that N protein remarkably inhibited poly(I·C)-induced phosphorylation of IRF3 (Fig. 4D). These results indicated that PPRV N protein abrogated IRF3 phosphorylation.

### PPRV N protein blocks IRF3 nuclear translocation.

**FIG 5:**
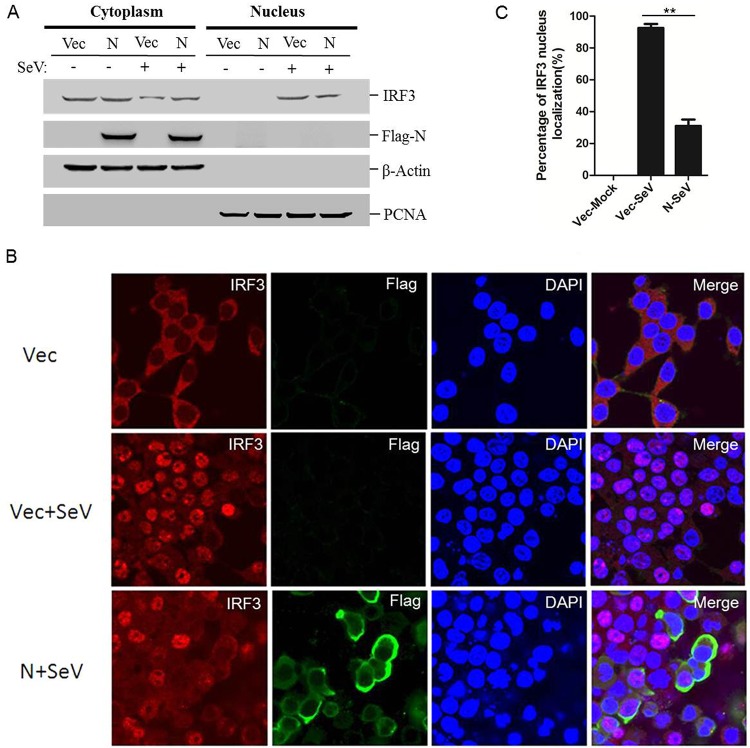

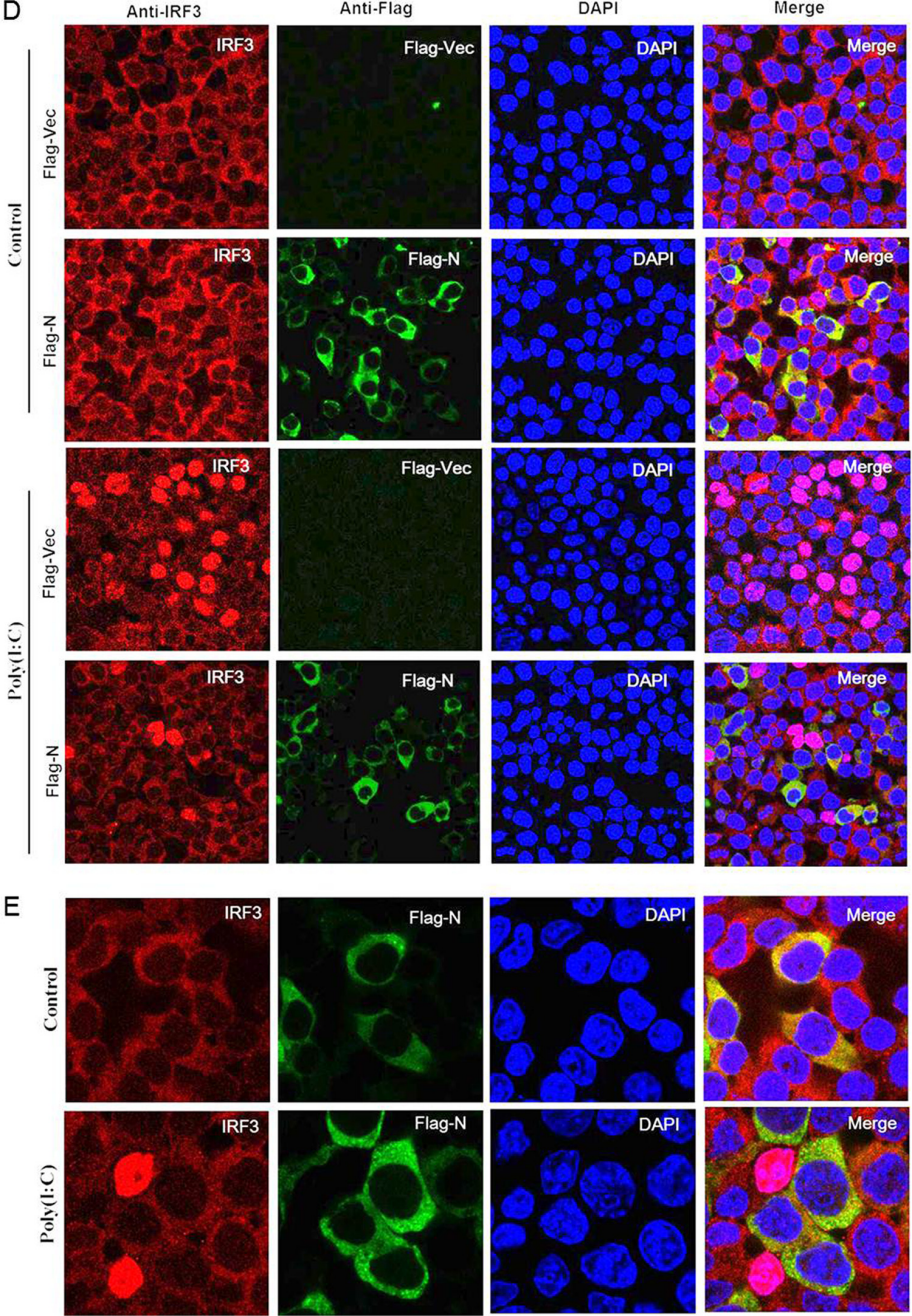
PPRV N protein colocalizes with IRF3 and inhibits IRF3 nuclear translocation. (A) HEK-293T cells were transfected with empty vector- or Flag-N-expressing plasmids for 24 h and then mock infected or infected with SeV for 16 h. Cytoplasmic and nuclear extracts were prepared and subjected to Western blotting. Expression of IRF3, N protein, β-actin, and proliferating cell nuclear antigen (PCNA) was detected with the appropriate antibodies. HEK-293T cells were transfected with empty vector- or Flag-N-expressing plasmids for 24 h and then mock infected or infected with SeV for 16 h. (B) The subcellular localization of IRF3 and N protein was analyzed by IFA. (C) The percentages of cells in which IRF3 localized to the nucleus were evaluated by counting 100 cells in randomly selected visual fields. (D and E) HEK-293T cells were transfected with empty vector- or Flag-N-expressing plasmids for 24 h and then transfected with poly(I·C) for 24 h. The subcellular localization of IRF3 and N protein was analyzed by IFA.

IRF3, a transcription factor involved in type I IFN production and ISG expression, plays its regulatory role after it is translocated into the nucleus (18). The phosphorylation of IRF3 causes its nuclear translocation. Our results showed that PPRV N protein interacts with IRF3 and inhibits IRF3 phosphorylation. To investigate the effects of N protein on the nuclear translocation of IRF3, HEK-293T cells were transfected with Flag-N-expressing plasmids and then infected with SeV. Nuclear and cytoplasmic extractions of endogenous IRF3 were performed, and the amounts of IRF3 in the nucleus and cytoplasm were analyzed. N protein inhibited SeV-induced nuclear translocation of IRF3 (Fig. 5A). The nuclear translocation status of IRF3 was also analyzed with an IFA. As expected, SeV infection induced significant nuclear translocation of IRF3 compared with that in mock-infected cells. However, this translocation was considerably reduced in the N protein-expressing cells (Fig. 5B). The main IRF3 nuclear location of cells was evaluated by counting 100 cells in randomly selected visual fields. In the mock-infected cells, IRF3 was localized predominately in the cytoplasm. After SeV infection, IRF3 translocated to the nucleus in approximately 94% of the cells. In the N protein-expressing cells, SeV-induced nuclear translocation of IRF3 was reduced to approximately 32% (Fig. 5C). The nuclear translocation status of IRF3 in poly(I·C)-transfected cells was further evaluated, which also showed that N protein significantly suppressed IRF3 nuclear transportation (Fig. 5D). In addition, the colocalization of N and endogenous IRF3 was also clearly observed (Fig. 5E). These results suggested that PPRV N protein blocks the nuclear translocation of IRF3 to prevent type I IFN production.

### PPRV N protein impairs the interaction of IRF3 with TBK1.

**FIG 6:**
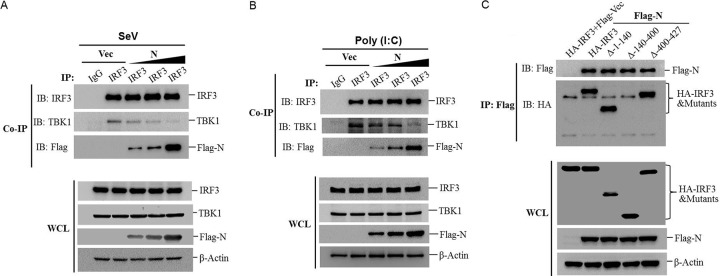
PPRV N protein disrupts TBK1-IRF3 complex formation. (A) HEK-293T cells were transfected with empty vector or increasing amounts of Flag-N-expressing plasmids (1, 2, or 5 μg) for 24 h and then infected with SeV for 16 h. The cells were lysed and immunoprecipitated with IRF3. The WCLs and IP complexes were analyzed by Western blotting using anti-IRF3, anti-TBK1, anti-Flag, and anti-β-actin antibodies. (B) HEK-293T cells were transfected with empty vector or increasing amounts of Flag-N-expressing plasmids (1, 2, or 5 μg) for 24 h and then transfected with poly(I·C) for 24 h. The cells were lysed and immunoprecipitated with IRF3. The WCLs and IP complexes were analyzed by Western blotting using anti-IRF3, anti-TBK1, anti-Flag, and anti-β-actin antibodies. (C) HEK-293T cells were cotransfected with equal amounts of empty vector- or Flag-N-expressing plasmids and HA-IRF3 or HA-tagged IRF3 truncated mutants expressing plasmids (HA-IRF3-Δ-1-140, HA-IRF3-Δ-140-400, or HA-IRF3-Δ-400-427) for 48 h. The cells were then lysed and immunoprecipitated with an anti-Flag antibody. The WCLs and IP complexes were analyzed by Western blotting using an anti-HA, anti-Flag, or anti-β-actin antibody.

The interaction between TBK1 and IRF3 is critical for phosphorylation of IRF3 and type I IFN production (19). Our initial results showed that N protein interacted with IRF3 and suppressed its phosphorylation. Therefore, it was reasonable to infer that N protein disrupted the interaction between TBK1 and IRF3. To test this hypothesis, HEK-293T cells were transfected with increasing amounts of Flag-N-expressing plasmids and then infected with SeV. A Co-IP assay was performed with the anti-IRF3 antibody, and the expression of TBK1, IRF3, and Flag-N was detected by Western blotting. As shown in Fig. 6A, both TBK1 and N protein were pulled down by IRF3. However, the amounts of TBK1 that coprecipitated with IRF3 gradually decreased as the expression of N protein increased. Similarly, we also investigated the effect of N protein on TBK1-IRF3 interaction in poly(I·C)-transfected cells. N protein significantly disrupted the poly(I·C)-induced interaction between TBK1 and IRF3 in a dose-dependent manner (Fig. 6B). In addition, the region of IRF3 crucial for the interaction between IRF3 and N protein was investigated, which showed that the region 140-400 is crucial for the IRF3-N interaction (Fig. 6C). These results suggested that PPRV N protein disrupted the interaction between IRF3 and TBK1 by targeting IRF3.

### The amino acid 106-210 region of PPRV N protein is essential for blocking IRF3 nuclear translocation and suppressing IFN-β promoter activation.

**FIG 7:**
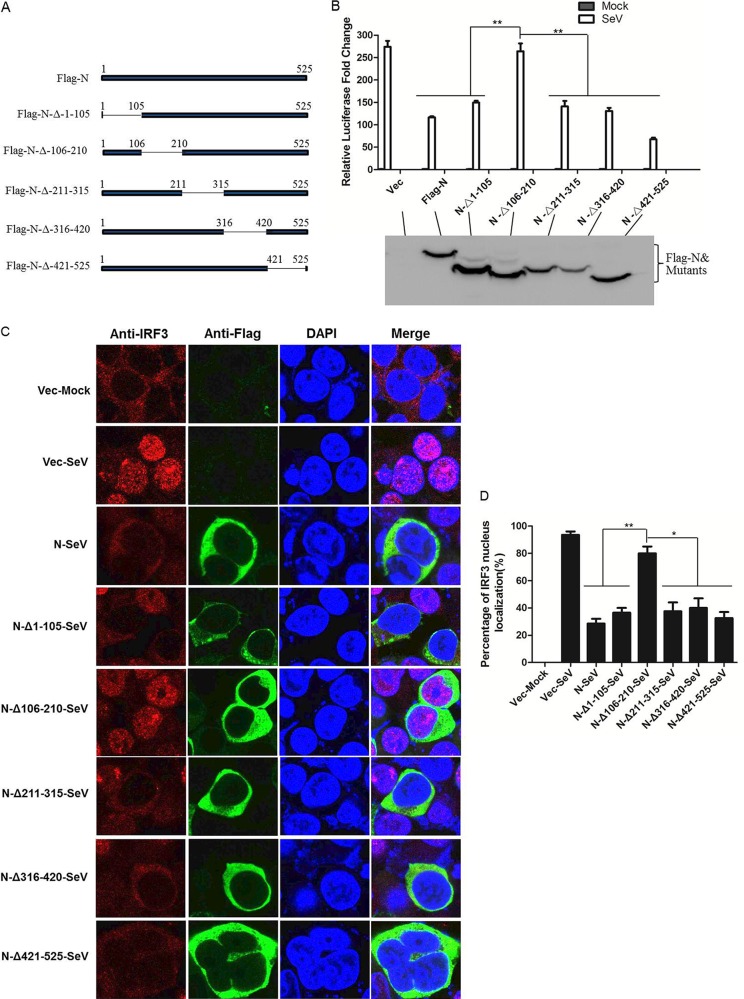
Effects of PPRV N mutants on SeV-induced IFN-β promoter activation and IRF3 nuclear translocation. (A) Schematic representation of a series of Flag-tagged truncated N constructs. (B) HEK-293T cells was cotransfected with empty vector, Flag-N-expressing plasmid, or the indicated PPRV N mutant-expressing plasmids and IFN-β promoter-driven reporter plasmids for 24 h and then infected with SeV for another 16 h. The luciferase activity was measured with a dual-luciferase assay. Expression of Flag-tagged N protein and the mutant proteins was evaluated by Western blotting. The subcellular localization of IRF3, Flag-N, and Flag-N mutants was determined with IFA (C), and the steady state of IRF3 localization was determined as for Fig. 5C (D).

N protein is the most abundant viral protein in PPRV-infected cells and plays important functions in PPRV replication. To investigate which region of N protein was responsible for the suppression of type I IFN production and IRF3 nuclear translocation, five N truncated mutants were constructed (Fig. 7A). HEK-293T cells were transfected with plasmids expressing various Flag-tagged N truncated mutants or full-length N protein, IFN-β promoter-driven luciferase reporter plasmid, and the internal control pRL-TK reporter plasmid and then infected with SeV. The luciferase activity was detected and analyzed. The deletion of the 106-210 amino acid region of N protein significantly abrogated its suppressive effect on SeV-induced activation of IFN-β promoter. However, the deletion of the 1-105, 211-315, 316-420, or 421-525 amino acid region of N protein had no effect on the N protein-mediated suppression of IFN-β promoter activation (Fig. 7B). The effects of these N protein truncation mutants on the nuclear translocation of IRF3 were also examined. HEK-293T cells were transfected with plasmids expressing various Flag-tagged N truncated mutants or full-length N protein and then infected with SeV. The nuclear translocation status of endogenous IRF3 was analyzed with an IFA. As shown in Fig. 7C, the N protein mutant in which amino acids 106 to 210 were deleted had a significantly reduced inhibitory effect on the nuclear translocation of IRF3. To have a quantitative evaluation of the nuclear translocation level of IRF3, the percentage of cells in which IRF3 localized to the nucleus was analyzed by counting 100 cells in randomly selected visual fields. The N protein mutants with the deletion of the 1-105, 211-315, 316-420, or 421-525 amino acid region showed similar rates of inhibition as that caused by the full-length N protein (Fig. 7D). This indicated that amino acids 106 to 210 of the PPRV N protein were involved in blocking the nuclear translocation of IRF3 and suppressing IFN production.

## DISCUSSION

The genus *Morbillivirus* includes MV, PPRV, canine distemper virus, and rinderpest virus. These viruses are highly contagious and cause severe diseases in various animals (20). PPRV is responsible for an acute disease in ruminants, which is widespread in developing countries and threatens developed countries despite vaccination (3). It has long been known that PPRV infections are associated with acute immunosuppression in their natural hosts, causing leukopenia, lymphopenia, and a reduced immune response (4, 9). Here we have shown that infection with PPRV significantly suppressed type I IFN production. Whether the abrogation of type I IFN production is responsible for the acute course of the infection and is associated with immunosuppression in the natural host remain unknown. The majority of morbilliviruses can antagonize host antiviral activities, but the mechanisms and proteins involved differ among the viruses (21–24). N protein, the most common viral protein, plays significant roles in suppressing host antiviral functions. For instance, the N proteins of MV and PPRV bind the peripheral blood lymphocytes of their natural host and suppress the cellular immune response (7). The MV N protein inhibits the inflammatory reaction by interacting with Fcγ receptor to perturb the host immune response (25). However, the role of N protein in the innate immune response has not been fully investigated.

In the present study, we demonstrated, for the first time, that PPRV N protein suppressed type I IFN production and inhibited the expression of various antiviral ISGs. Type I IFN triggers cellular antiviral responses through induction of ISG expression (26), and both type I IFN and ISGs are critical to the antiviral defenses (27). To support viral replication in their host cells, viruses have evolved different antagonistic strategies that affect the activation of important signaling proteins or inhibit IFN production. Zika virus degrades the IFN-regulated transcriptional activator STAT2 in humans to inhibit type I IFN signaling (28). Rotavirus NSP1 degrades IFN regulatory factors IRF3, IRF5, and IRF7 to antagonize type I IFN pathway activation and overcome the type I IFN response (29). The V protein of human parainfluenza virus 2 induces the proteolytic degradation of STAT2 to suppress ISG expression (30).

In this study, IRF3 was identified as a target of PPRV N protein, through which it suppressed type I IFN production. IRF3, a key transcription factor, plays crucial roles in the induction of type I IFN synthesis and is required for the expression of many genes involved in the innate immune responses (31). Therefore, IRF3 becomes the target of many different viral IFN antagonists (32–35). Different antagonistic mechanisms targeting IRF3 have been identified in various viruses. Foot-and-mouth disease virus and Senecavirus A reduce the abundance of IRF3 with a viral protease to attenuate type I IFN pathway activation (36, 37). Varicella zoster virus abrogates the IRF3-mediated innate immune response by degrading activated IRF3 (38). The Thogoto virus nonessential accessory protein ML antagonizes host IFN signaling by specifically inhibiting the interaction between IRF3 and the transcriptional coactivator CREB-binding protein (CBP) in the nucleus (32). Ebola virus blocks the dimerization and phosphorylation of IRF3 to interrupt IRF3 function and inhibit the antiviral response (34). Our data demonstrated that PPRV N protein interacted with IRF3 and blocked the interaction between TBK1 and IRF3, which was crucial for the activation of IRF3 and production of type I IFN. The N protein-mediated inhibitory effect on the TBK1-IRF3 interaction blocked type I IFN synthesis and the downstream signaling in the type I IFN pathway. N protein did not interact with TBK1, and so the interaction between N protein and IRF3 might block the interaction between TBK1 and IRF3, resulting in the suppression of both IRF3 activation and IFN synthesis.

The deletion of the 106-210 region of N protein almost completely abrogated the suppressive effect of N protein on type I IFN production and IRF3 nuclear transportation, suggesting an important antagonistic role of this region and implying a potential role of this region for the interaction between PPRV N protein and host IRF3 protein. PPRV N protein contains an N-terminal core domain of approximately 400 residues and a C-terminal tail domain (39). The N-terminal core domain is responsible for RNA encapsidation and is involved in the formation of viral helical nucleocapsid that is important for PPRV replication (40). The C-terminal tail domain is identified as a determinant for viral pathogenesis that is involved in the regulation of the activity of viral transcriptase (39). The region of 106 to 210 is located in the N core domain. This suggests that the N core domain performs multiple functions, not only encapsidating the viral RNA but also playing an antagonistic role against host antiviral response. The 106-210 region of the N core domain might be a novel target to engineer gradual virus attenuation for next-generation PPRV vaccine design using the reverse genetic system. The 140-400 region of IRF3 was identified as critical region for the interaction between N protein and IRF3. IRF3 contains a conserved DNA binding domain (DBD), an IRF association domain (IAD), and a C-terminal autoinhibition element (AIE) (41). The 140-400 region includes the IAD domain that is significantly involved in the formation of the IRF3 homodimer (41, 42). In addition, the sequential phosphorylation of distinct sites (Ser385/Ser386 → Ser396 → Thr390 → Ser396) in IRF3 is instrumental for IRF3 activation (43), and these sites are located in the 140-400 region. PPRV N protein might interact with several sites of the 140-400 region to prevent the phosphorylation of IRF3 induced by TBK1 kinase and then block the dimerization of IRF3 and impair IFN production.

**FIG 8:**
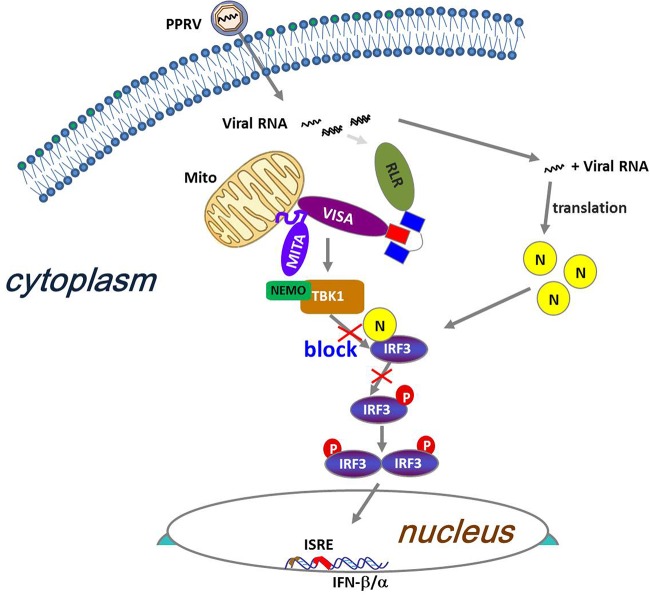
Proposed working model of how PPRV N protein negatively regulates type I IFN production. PPRV N protein negatively regulates type I IFN production by impairing the interaction between TBK1 and IRF3 to inhibit the phosphorylation of IRF3 and prevent nuclear translocation of IRF3.

In summary, our data identified the antagonistic mechanism by which the PPRV N protein blocked type I IFN production (Fig. 8). We have demonstrated that the PPRV N protein interfered with the formation of the TBK1-IRF3 complex by targeting IRF3. These findings suggested a novel strategy by which PPRV subverted type I IFN production and evaded host innate immune response and also provided us with new insight into the pathogenesis of PPRV.

## MATERIALS AND METHODS

### Cells and viruses.

Vero (an African green monkey kidney cell line) cells and HEK-293T (a human embryonic kidney cell line) cells were cultured in Dulbecco’s modified Eagle’s medium (DMEM) (Gibco) supplemented with 10% fetal bovine serum (FBS) (Gibco) in 5% CO_2_ at 37°C. The primary goat fibroblasts and macrophages were isolated and conserved by our lab. The PPRV strain China/XJBZ/2015 was isolated by our lab previously and was used in this study, and the viruses were propagated in Vero cells (44). SeV, a model virus extensively used to induce IFN production and activate type I IFN signaling in cell culture was propagated in 9-day-old specific-pathogen-free chicken embryos; the strain used and infection method have been described previously (45).

### Antibodies and reagents.

Mouse monoclonal antibodies against Flag were purchased from Sigma. Mouse monoclonal antibodies against β-actin, mouse anti-IRF3 antibodies, and rabbit monoclonal antibodies against Flag were purchased from Santa Cruz Biotechnology. Mouse monoclonal antibodies against HA were purchased from BioLegend. Rabbit anti-IRF3 and anti-phospho-IRF3 and mouse anti-PCNA antibodies were purchased from Cell Signaling Technology. Goat anti-mouse or rabbit IgG(H+L) secondary antibodies were purchased from Thermo Fisher Scientific. Goat anti-mouse IgG heavy- or light-chain-specific secondary antibodies were purchased from Abbkine Scientific Co. The Alexa Fluor secondary antibodies for IFA were purchased from Life Technologies. 4',6-Diamidino-2-phenylindole (DAPI) was purchased from Roche. Rabbit anti-N protein polyclonal antibody was prepared by our lab.

### Construction of eukaryotic expression plasmids and cell transfection.

Each of the PPRV full-length viral coding region fragments was amplified and cloned into p3xFLAG-CMV-7.1 vector (Sigma-Aldrich) to establish the eukaryotic plasmids expressing Flag-tagged viral proteins. A series of Flag-tagged PPRV truncated N mutants were generated by a conventional PCR method using the Flag-N plasmids as the template. The IFN-β promoter luciferase reporter plasmids, pRL-TK plasmid, and various HA-tagged plasmids used in this study were kindly provided by Hongbing Shu (Wuhan University, China) (46). All the constructed plasmids were sequenced and the correct insertion of each gene was verified. The DNA plasmids were transfected into the cells using the transfection reagent Lipofectamine 3000 (Invitrogen) according to the manufacturer’s instructions.

### Dual-luciferase reporter assay.

HEK-293T cells were cultured in 24-well plates, and the monolayer cells were transfected with 0.2 μg of Flag vector-, Flag-N-, Flag-M-, Flag-C-, Flag-F-, Flag-H-, or Flag-P-expressing plasmids, 0.1 μg of IFN-β promoter-driven luciferase reporter plasmid, and 0.01 μg of internal control pRL-TK reporter plasmid (normalization of transfection efficiency). The empty vector plasmids were used in the whole transfection process to ensure the cells received the same amounts of total plasmids. The transfected cells were mock infected or infected with SeV at 24 hpt (100 hemagglutinating activity units [HAU]/ml). The cells were lysed at 16 h postinfection (hpi), and the luciferase activities were measured using a dual-luciferase reporter assay kit (Promega) according to the standard protocols provided by the manufacturer. As for the adaptor molecule-induced IFN-β promoter activation assay, the HEK-293T cells were cotransfected with 0.1 μg of the IFN-β promoter-driven luciferase reporter plasmids, 0.01 μg of pRL-TK reporter plasmids, 0.1 μg of the indicated adaptor plasmids or vector plasmid, and 0.2 μg of Flag-vector- or Flag-N-expressing plasmids. The dual-luciferase activities were measured and analyzed at 26 hpt. All experiments were performed in triplicates and repeated at least three times. The results represent the means and standard deviations of data from three independent experiments.

### Real-time qPCR.

Total RNAs were isolated using TRIzol reagent (Invitrogen) and reverse transcribed into cDNA using the M-MLV reverse transcriptase (Invitrogen) system according to the manufacture’s protocols. The SYBR Premix *Ex Taq* reagents (TaKaRa) were used to quantify the RNA copy numbers. The glyceraldehyde-3-phosphate dehydrogenase (GAPDH) gene, the most commonly used housekeeping gene, was used as an internal control. Relative abundance of mRNA transcripts was analyzed and calculated by the threshold cycle method (2^ΔΔ^*^CT^*).

### IFA and confocal imaging.

HEK-293T cells were seeded into Nunc glass-bottom dishes, cultured overnight, and transfected with 2 μg of HA vector or HA-IRF3 and 2 μg of Flag vector or Flag-N plasmids for 36 h. As for the nuclear translocation assay, the monolayer HEK-293T cells were transfected with Flag vector or Flag-N plasmids, and the cells were then mock infected or infected with SeV at 24 hpt for 16 h. All the collected cells were fixed with 4% paraformaldehyde in phosphate-buffered saline (PBS) at 4°C for 2 h. After 3 washes with ice-cold PBS, the cells were permeabilized with 0.2% Triton X-100 for 10 min; 5% bovine serum albumin (BSA) in PBS was used as blocking buffer. After blocking at 37°C for 1 h, the cells were incubated with the proper antibodies as described previously (45). The stained cells were examined with a Nikon eclipse 80i fluorescence microscope. The images were analyzed with NIS Elements F 2.30 software.

### Co-IP and Western blotting.

Co-IP assays were performed as described previously (47). Briefly, the cells grown in the 10-cm dishes were transfected with the various plasmids for 48 h, and the transfected cells were collected and lysed for immunoprecipitation. A 50% (vol/vol) slurry of GammaBind G Plus-Sepharose (GE Health Care Life Sciences) and proper antibodies was used to immunoprecipitate the interacted proteins. For Western blotting, the lysed samples were boiled with sample buffer (2% SDS, 10% glycerol, 60 mM Tris [pH 6.8], 5% β-mercaptoethanol, and 0.01% bromophenol blue) for 5 min to denature the proteins, followed by a 10-min centrifugation at 20,000 × *g* at 4°C. The supernatants were then analyzed by SDS-PAGE, and Western blotting was performed with the appropriate antibody as described previously (48).

### Preparation of nuclear and cytoplasmic extracts.

Nuclear and cytoplasmic extracts were separated using a nuclear/cytosol fractionation kit (BioVision) according to the manufacturer’s instructions. The purity of cytoplasmic and nuclear extracts was evaluated by immunoblotting with anti-β-actin and anti-PCNA antibodies, respectively. The prepared nuclear and cytoplasmic extracts were subjected to Western blotting.

### Statistical analysis.

All data are presented as mean values ± standard errors (SEs) from three independent experiments. Two-tail Student's *t* tests were used to analyze the significance of the data. Differences were considered to be statistically significant if the *P* value was <0.05, and a *P* value of <0.01 was considered highly significant.
